# 4-[(2-Hy­droxy­naphthalen-1-yl)(morpholin-4-yl)meth­yl]benzonitrile

**DOI:** 10.1107/S160053681104997X

**Published:** 2011-11-30

**Authors:** Xin-Yuan Chen, Min-Min Zhao, Xu Qian, Shao-Gang Hou

**Affiliations:** a Ordered Matter Science Research Center, Department of Chemistry and Chemical Engineering, Southeast University, Nanjing 210096, People’s Republic of China

## Abstract

The title compound, C_22_H_20_N_2_O_2_, was synthesized *via* a multicomponent reaction using naphthalen-2-ol, morpholine and 4-formyl­benzonitrile. The dihedral angle between the naphthalene ring system and the benzene ring is 81.25 (10)°. The morpholine ring adopts a chair conformation. The mol­ecular conformation is stabilized by intra­molecular O—H⋯N and C—H⋯O hydrogen bonds. In the crystal, inter­molecular C—H⋯N hydrogen bonds link mol­ecules into helical chains running parallel to the *c* axis.

## Related literature

For background to multi-component reactions, see: Devi & Bhuyan (2004 ▶); Domling & Ugi (2000 ▶). Hulme & Gore (2003 ▶); Ugi (1962 ▶). For ring puckering parameters, see: Cremer & Pople (1975 ▶).
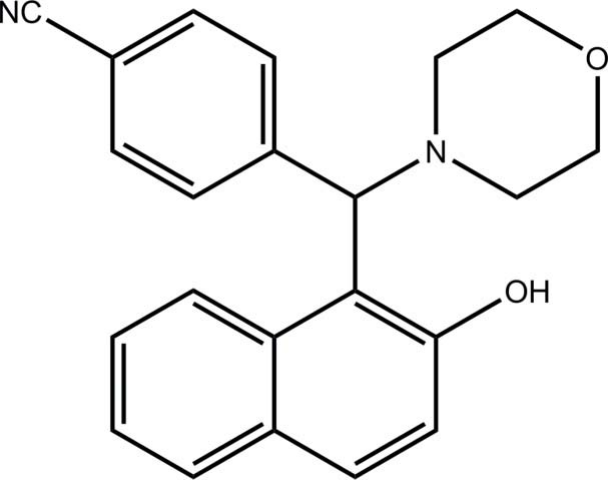


## Experimental

### 

#### Crystal data

C_22_H_20_N_2_O_2_
*M* *_r_* = 344.40
Trigonal, 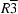

*a* = 18.294 (3) Å
*c* = 28.738 (6) Å
*V* = 8329 (4) Å^3^
*Z* = 18
Mo *K*α radiation
μ = 0.08 mm^−1^
*T* = 298 K
0.20 × 0.15 × 0.10 mm

#### Data collection

Rigaku Mercury2 diffractometer
Absorption correction: multi-scan (*CrystalClear*; Rigaku, 2005 ▶) *T*_min_ = 0.910, *T*_max_ = 1.000
24077 measured reflections
3326 independent reflections
1786 reflections with *I* > 2σ(*I*)
*R*_int_ = 0.138

#### Refinement

*R*[*F* ^2^ > 2σ(*F* ^2^)] = 0.081
*wR*(*F* ^2^) = 0.210
*S* = 1.03
3326 reflections
235 parameters
7 restraints
H-atom parameters constrained
Δρ_max_ = 0.22 e Å^−3^
Δρ_min_ = −0.27 e Å^−3^

### 

Data collection: *CrystalClear* (Rigaku, 2005 ▶); cell refinement: *CrystalClear*; data reduction: *CrystalClear*; program(s) used to solve structure: *SHELXS97* (Sheldrick, 2008 ▶); program(s) used to refine structure: *SHELXL97* (Sheldrick, 2008 ▶); molecular graphics: *SHELXTL* (Sheldrick, 2008 ▶); software used to prepare material for publication: *SHELXTL*.

## Supplementary Material

Crystal structure: contains datablock(s) I, global. DOI: 10.1107/S160053681104997X/rz2668sup1.cif

Structure factors: contains datablock(s) I. DOI: 10.1107/S160053681104997X/rz2668Isup2.hkl

Supplementary material file. DOI: 10.1107/S160053681104997X/rz2668Isup3.cml

Additional supplementary materials:  crystallographic information; 3D view; checkCIF report

## Figures and Tables

**Table 1:** Hydrogen-bond geometry (Å, °)

*D*—H⋯*A*	*D*—H	H⋯*A*	*D*⋯*A*	*D*—H⋯*A*
O1—H1⋯N1	0.85	1.82	2.601 (4)	151
C21—H21*A*⋯O1	0.93	2.54	3.300 (4)	139
C7—H7*A*⋯N2^i^	0.93	2.44	3.327 (9)	160

Symmetry code: (i) 
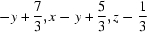
.

**Fig. 1.**
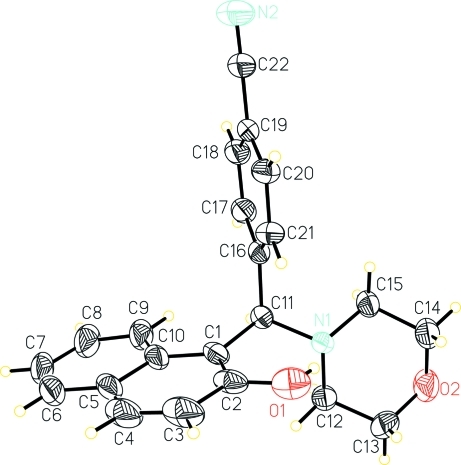
The molecular structure of the title compound showing displacement ellipsoids drawn at the 30% probability level.

## supplementary crystallographic information

### Comment

Multi-component reactions (MCRs) (Hulme & Gore, 2003; Ugi, 1962) involving at least three starting materials in a one-pot reaction have attracted considerable attention in terms of saving both energy and raw materials (Devi & Bhuyan, 2004). Compared to conventional multi-step organic syntheses, MCRs have advantages that include the simplicity of a one-pot procedure and the buildup of complex molecules (Domling & Ugi, 2000). We report here the synthesis and crystal structure of the title compound, 4-4-[(2-hydroxynaphthalen-1-yl)(morpholino)methyl]benzonitrile.

In the title compound (Fig. 1) bond lengths and angles have normal values. The dihedral angle between the naphthalene ring system and the benzene ring is 81.25 (10)°. The morpholine ring (N1/C12/C13/O2/C14/C15) assumes a boat conformation, with puckering parameters <i<Q, θ and φ (Cremer & Pople, 1975) of 0.559 (4) Å, 179.3 (4)° and -159 (4)°, respectively. The molecular conformation is stabilized by intramolecular O—H···N and C—H···O hydrogen bonds (Table 1). In the crystal structure, molecules are linked into helical chains parallel to the c axis by intermolecular C—H···N hydrogen bonds.

### Experimental

A dry 100 ml flask was charged with 4-formylbenzonitrile (15 mmol), naphthalen-2-ol (15 mmol) and morpholine (15 mmol). The mixture was stirred at 373 K for 12 h, then ethanol (15 ml) was added. After heating under reflux for 1 h, the precipitate was filtrated out and washed with ethanol (10 ml × 3) to give the title compound. Colourless crystals were obtained by slow evaporation of a dichloromethane solution.

### Refinement

All the H atoms attached to C atoms were situated into the idealized positions and treated as riding, with C–H = 0.93 Å (aromatic), 0.97 Å (methylene) and 0.98 Å (methine), and with *U_iso_*(H) = 1.2*U_eq_*(C). The hydroxyl H atom was located in a difference Fourier map and refined as riding, with O—H = 0.82 Å and with *U_iso_*(H) = 1.5*U_eq_*(O). Restraints (SIMU and DELU) were used for stabilizing the refinement of atoms C5 and C6. The quality of the crystal available was not optimal and it was weakly diffracting, with no significant data obtained beyond θ = 20°. Although recrystallization was attempted repeatedly, no better crystals could be obtained. This could account for the rather high *R*_int_ value (0.138) and for the poor precision of the analysis.

### Crystal data

**Table:**

C_22_H_20_N_2_O_2_	*D*_x_ = 1.236 Mg m^−^^3^
*M**_r_* = 344.40	Mo *K*α radiation, λ = 0.71073 Å
Trigonal, *R*3	Cell parameters from 3326 reflections
Hall symbol: -R 3	θ = 3.1–25.2°
*a* = 18.294 (3) Å	µ = 0.08 mm^−^^1^
*c* = 28.738 (6) Å	*T* = 298 K
*V* = 8329 (4) Å^3^	Block, colourless
*Z* = 18	0.20 × 0.15 × 0.10 mm
*F*(000) = 3276	

### Data collection

**Table:**

Rigaku Mercury2 diffractometer	3326 independent reflections
Radiation source: fine-focus sealed tube	1786 reflections with *I* > 2σ(*I*)
graphite	*R*_int_ = 0.138
Detector resolution: 13.6612 pixels mm^-1^	θ_max_ = 25.2°, θ_min_ = 3.1°
CCD profile fitting scans	*h* = −21→21
Absorption correction: multi-scan (*CrystalClear*; Rigaku, 2005)	*k* = −21→21
*T*_min_ = 0.910, *T*_max_ = 1.000	*l* = −34→34
24077 measured reflections	

### Refinement

**Table:**

Refinement on *F*^2^	Primary atom site location: structure-invariant direct methods
Least-squares matrix: full	Secondary atom site location: difference Fourier map
*R*[*F*^2^ > 2σ(*F*^2^)] = 0.081	Hydrogen site location: inferred from neighbouring sites
*wR*(*F*^2^) = 0.210	H-atom parameters constrained
*S* = 1.03	*w* = 1/[σ^2^(*F*_o_^2^) + (0.0825*P*)^2^ + 7.1283*P*] where *P* = (*F*_o_^2^ + 2*F*_c_^2^)/3
3326 reflections	(Δ/σ)_max_ < 0.001
235 parameters	Δρ_max_ = 0.22 e Å^−^^3^
7 restraints	Δρ_min_ = −0.27 e Å^−^^3^

### Special details

**Table:**

Geometry. All esds (except the esd in the dihedral angle between two l.s. planes) are estimated using the full covariance matrix. The cell esds are taken into account individually in the estimation of esds in distances, angles and torsion angles; correlations between esds in cell parameters are only used when they are defined by crystal symmetry. An approximate (isotropic) treatment of cell esds is used for estimating esds involving l.s. planes.
Refinement. Refinement of F^2^ against ALL reflections. The weighted R-factor wR and goodness of fit S are based on F^2^, conventional R-factors R are based on F, with F set to zero for negative F^2^. The threshold expression of F^2^ > 2sigma(F^2^) is used only for calculating R-factors(gt) etc. and is not relevant to the choice of reflections for refinement. R-factors based on F^2^ are statistically about twice as large as those based on F, and R- factors based on ALL data will be even larger.

### Fractional atomic coordinates and isotropic or equivalent isotropic displacement parameters (Å^2^)

**Table:**

	*x*	*y*	*z*	*U*_iso_*/*U*_eq_	
C1	0.6590 (2)	0.9543 (2)	−0.01901 (11)	0.0550 (9)	
N1	0.58212 (15)	0.86806 (15)	0.04966 (8)	0.0455 (7)	
O1	0.51037 (19)	0.90933 (18)	−0.01526 (10)	0.0841 (9)	
H1	0.5181	0.8920	0.0108	0.126*	
C2	0.5846 (3)	0.9432 (2)	−0.03881 (13)	0.0713 (12)	
N2	0.7870 (3)	1.3181 (3)	0.14213 (18)	0.146 (2)	
O2	0.48604 (18)	0.71036 (17)	0.09733 (11)	0.0913 (10)	
C3	0.5833 (5)	0.9667 (3)	−0.08470 (18)	0.110 (2)	
H3A	0.5334	0.9598	−0.0972	0.132*	
C4	0.6527 (7)	0.9991 (4)	−0.11117 (19)	0.140 (3)	
H4A	0.6504	1.0157	−0.1414	0.168*	
C5	0.7294 (5)	1.0085 (3)	−0.09428 (18)	0.119 (2)	
C6	0.8038 (6)	1.0382 (4)	−0.1215 (2)	0.145 (3)	
H6A	0.8020	1.0527	−0.1524	0.174*	
C7	0.8752 (5)	1.0465 (4)	−0.1058 (3)	0.160 (4)	
H7A	0.9223	1.0672	−0.1250	0.192*	
C8	0.8794 (4)	1.0236 (3)	−0.0596 (2)	0.131 (2)	
H8A	0.9289	1.0275	−0.0484	0.158*	
C9	0.8103 (3)	0.9956 (2)	−0.03107 (17)	0.0894 (15)	
H9A	0.8146	0.9826	−0.0003	0.107*	
C10	0.7325 (3)	0.9858 (2)	−0.04718 (13)	0.0728 (12)	
C11	0.66336 (19)	0.93938 (19)	0.03258 (10)	0.0459 (8)	
H11A	0.7076	0.9249	0.0374	0.055*	
C12	0.5716 (2)	0.7879 (2)	0.03122 (13)	0.0606 (10)	
H12A	0.6184	0.7811	0.0413	0.073*	
H12B	0.5719	0.7894	−0.0025	0.073*	
C13	0.4897 (3)	0.7141 (2)	0.04806 (16)	0.0834 (13)	
H13A	0.4428	0.7192	0.0363	0.100*	
H13B	0.4842	0.6621	0.0360	0.100*	
C14	0.4948 (3)	0.7858 (2)	0.11521 (14)	0.0771 (12)	
H14A	0.4925	0.7828	0.1489	0.092*	
H14B	0.4480	0.7922	0.1045	0.092*	
C15	0.5765 (2)	0.8617 (2)	0.10044 (12)	0.0584 (9)	
H15A	0.5798	0.9124	0.1132	0.070*	
H15B	0.6235	0.8572	0.1126	0.070*	
C16	0.6885 (2)	1.0214 (2)	0.05850 (10)	0.0478 (8)	
C17	0.7626 (2)	1.0606 (2)	0.08376 (12)	0.0621 (10)	
H17A	0.7954	1.0350	0.0863	0.075*	
C18	0.7886 (2)	1.1370 (3)	0.10521 (13)	0.0733 (12)	
H18A	0.8395	1.1633	0.1214	0.088*	
C19	0.7396 (3)	1.1747 (2)	0.10287 (12)	0.0651 (11)	
C20	0.6641 (2)	1.1355 (2)	0.07818 (12)	0.0626 (10)	
H20A	0.6302	1.1600	0.0767	0.075*	
C21	0.6400 (2)	1.0603 (2)	0.05593 (11)	0.0554 (9)	
H21A	0.5902	1.0349	0.0388	0.067*	
C22	0.7657 (3)	1.2544 (3)	0.12509 (16)	0.0976 (16)	

### Atomic displacement parameters (Å^2^)

**Table:**

	*U*^11^	*U*^22^	*U*^33^	*U*^12^	*U*^13^	*U*^23^
C1	0.086 (3)	0.047 (2)	0.036 (2)	0.036 (2)	0.0023 (19)	−0.0008 (15)
N1	0.0493 (16)	0.0456 (16)	0.0418 (16)	0.0238 (13)	0.0012 (12)	−0.0033 (12)
O1	0.090 (2)	0.090 (2)	0.084 (2)	0.0538 (18)	−0.0321 (17)	−0.0075 (16)
C2	0.118 (4)	0.054 (2)	0.045 (2)	0.045 (3)	−0.022 (2)	−0.0062 (18)
N2	0.126 (4)	0.096 (3)	0.153 (4)	0.008 (3)	0.030 (3)	−0.067 (3)
O2	0.106 (2)	0.0635 (19)	0.092 (2)	0.0338 (17)	0.0362 (18)	0.0195 (16)
C3	0.189 (6)	0.092 (4)	0.060 (3)	0.077 (4)	−0.045 (4)	−0.004 (3)
C4	0.280 (10)	0.078 (4)	0.047 (4)	0.078 (5)	−0.024 (5)	0.001 (3)
C5	0.231 (6)	0.043 (2)	0.049 (3)	0.043 (3)	0.054 (3)	0.005 (2)
C6	0.250 (6)	0.058 (3)	0.066 (3)	0.031 (4)	0.070 (4)	−0.002 (2)
C7	0.193 (7)	0.068 (4)	0.144 (7)	0.008 (5)	0.123 (6)	−0.008 (4)
C8	0.129 (5)	0.080 (3)	0.154 (6)	0.028 (3)	0.087 (4)	−0.007 (3)
C9	0.099 (4)	0.064 (3)	0.095 (3)	0.034 (3)	0.051 (3)	0.004 (2)
C10	0.112 (4)	0.041 (2)	0.055 (3)	0.031 (2)	0.028 (2)	−0.0009 (18)
C11	0.0470 (19)	0.049 (2)	0.044 (2)	0.0261 (17)	0.0012 (15)	0.0000 (15)
C12	0.068 (2)	0.052 (2)	0.063 (2)	0.0313 (19)	0.0085 (19)	0.0001 (17)
C13	0.086 (3)	0.048 (2)	0.098 (4)	0.021 (2)	0.013 (3)	−0.004 (2)
C14	0.083 (3)	0.068 (3)	0.077 (3)	0.036 (2)	0.026 (2)	0.010 (2)
C15	0.063 (2)	0.063 (2)	0.049 (2)	0.032 (2)	0.0085 (17)	0.0072 (17)
C16	0.048 (2)	0.053 (2)	0.0362 (19)	0.0210 (17)	0.0011 (15)	0.0025 (15)
C17	0.051 (2)	0.073 (3)	0.055 (2)	0.025 (2)	−0.0007 (17)	−0.0086 (19)
C18	0.051 (2)	0.083 (3)	0.059 (3)	0.014 (2)	0.0010 (18)	−0.020 (2)
C19	0.068 (3)	0.053 (2)	0.045 (2)	0.009 (2)	0.0174 (19)	−0.0085 (17)
C20	0.074 (3)	0.054 (2)	0.059 (2)	0.032 (2)	0.002 (2)	−0.0073 (18)
C21	0.065 (2)	0.049 (2)	0.052 (2)	0.0287 (19)	−0.0093 (17)	−0.0094 (17)
C22	0.081 (3)	0.073 (3)	0.088 (3)	0.000 (2)	0.024 (2)	−0.033 (3)

### Geometric parameters (Å, °)

**Table:**

C1—C2	1.392 (5)	C9—H9A	0.9300
C1—C10	1.422 (5)	C11—C16	1.526 (4)
C1—C11	1.517 (4)	C11—H11A	0.9800
N1—C15	1.464 (4)	C12—C13	1.511 (5)
N1—C12	1.478 (4)	C12—H12A	0.9700
N1—C11	1.488 (4)	C12—H12B	0.9700
O1—C2	1.359 (5)	C13—H13A	0.9700
O1—H1	0.8517	C13—H13B	0.9700
C2—C3	1.391 (6)	C14—C15	1.505 (5)
N2—C22	1.137 (5)	C14—H14A	0.9700
O2—C14	1.405 (4)	C14—H14B	0.9700
O2—C13	1.418 (5)	C15—H15A	0.9700
C3—C4	1.337 (9)	C15—H15B	0.9700
C3—H3A	0.9300	C16—C17	1.381 (4)
C4—C5	1.410 (9)	C16—C21	1.389 (4)
C4—H4A	0.9300	C17—C18	1.377 (5)
C5—C6	1.423 (10)	C17—H17A	0.9300
C5—C10	1.425 (7)	C18—C19	1.379 (5)
C6—C7	1.317 (10)	C18—H18A	0.9300
C6—H6A	0.9300	C19—C20	1.391 (5)
C7—C8	1.403 (10)	C19—C22	1.437 (6)
C7—H7A	0.9300	C20—C21	1.374 (4)
C8—C9	1.373 (6)	C20—H20A	0.9300
C8—H8A	0.9300	C21—H21A	0.9300
C9—C10	1.420 (6)		
			
C2—C1—C10	118.9 (4)	N1—C12—H12A	109.5
C2—C1—C11	120.6 (3)	C13—C12—H12A	109.5
C10—C1—C11	120.3 (3)	N1—C12—H12B	109.5
C15—N1—C12	108.1 (3)	C13—C12—H12B	109.5
C15—N1—C11	113.5 (2)	H12A—C12—H12B	108.1
C12—N1—C11	109.2 (2)	O2—C13—C12	111.4 (3)
C2—O1—H1	107.0	O2—C13—H13A	109.4
O1—C2—C3	116.4 (5)	C12—C13—H13A	109.4
O1—C2—C1	123.0 (3)	O2—C13—H13B	109.4
C3—C2—C1	120.6 (5)	C12—C13—H13B	109.4
C14—O2—C13	109.8 (3)	H13A—C13—H13B	108.0
C4—C3—C2	121.1 (6)	O2—C14—C15	112.1 (3)
C4—C3—H3A	119.4	O2—C14—H14A	109.2
C2—C3—H3A	119.4	C15—C14—H14A	109.2
C3—C4—C5	121.6 (5)	O2—C14—H14B	109.2
C3—C4—H4A	119.2	C15—C14—H14B	109.2
C5—C4—H4A	119.2	H14A—C14—H14B	107.9
C4—C5—C6	124.1 (7)	N1—C15—C14	110.6 (3)
C4—C5—C10	118.2 (6)	N1—C15—H15A	109.5
C6—C5—C10	117.6 (8)	C14—C15—H15A	109.5
C7—C6—C5	124.0 (8)	N1—C15—H15B	109.5
C7—C6—H6A	118.0	C14—C15—H15B	109.5
C5—C6—H6A	118.0	H15A—C15—H15B	108.1
C6—C7—C8	119.3 (6)	C17—C16—C21	118.4 (3)
C6—C7—H7A	120.3	C17—C16—C11	120.1 (3)
C8—C7—H7A	120.3	C21—C16—C11	121.5 (3)
C9—C8—C7	119.9 (7)	C18—C17—C16	120.9 (4)
C9—C8—H8A	120.0	C18—C17—H17A	119.6
C7—C8—H8A	120.0	C16—C17—H17A	119.6
C8—C9—C10	121.9 (5)	C17—C18—C19	120.3 (3)
C8—C9—H9A	119.0	C17—C18—H18A	119.8
C10—C9—H9A	119.0	C19—C18—H18A	119.8
C9—C10—C1	123.4 (4)	C18—C19—C20	119.5 (3)
C9—C10—C5	117.2 (5)	C18—C19—C22	121.1 (4)
C1—C10—C5	119.4 (5)	C20—C19—C22	119.4 (4)
N1—C11—C1	111.2 (3)	C21—C20—C19	119.5 (4)
N1—C11—C16	112.3 (2)	C21—C20—H20A	120.2
C1—C11—C16	108.5 (2)	C19—C20—H20A	120.2
N1—C11—H11A	108.3	C20—C21—C16	121.4 (3)
C1—C11—H11A	108.3	C20—C21—H21A	119.3
C16—C11—H11A	108.3	C16—C21—H21A	119.3
N1—C12—C13	110.6 (3)	N2—C22—C19	179.0 (5)

### Hydrogen-bond geometry (Å, °)

**Table:**

*D*—H···*A*	*D*—H	H···*A*	*D*···*A*	*D*—H···*A*
O1—H1···N1	0.85	1.82	2.601 (4)	151.
C21—H21A···O1	0.93	2.54	3.300 (4)	139
C7—H7A···N2^i^	0.93	2.44	3.327 (9)	160

Symmetry codes: (i) −*y*+7/3, *x*−*y*+5/3, *z*−1/3.
